# Association between dietary fiber intake and post-stroke depression among US women: insights from the NHANES 2005–2018 cross-sectional study

**DOI:** 10.3389/fnut.2025.1586511

**Published:** 2025-07-29

**Authors:** Xueshan Jian, Shuyang Jian, Zhiru Zhang, Yuxuan Ye, Xiaona Tang, Rucheng Huang

**Affiliations:** ^1^The Seventh Clinical College of Guangzhou University of Chinese Medicine, Shenzhen, China; ^2^Department of Encephalopathy, Baoan District Hospital of Traditional Chinese Medicine, Shenzhen, China; ^3^Nursing Department, Baoan District Hospital of Traditional Chinese Medicine, Shenzhen, China

**Keywords:** dietary fiber, stroke, depression, post-stroke depression, logistic regression model, NHANES, cross-sectional study

## Abstract

**Background:**

Few studies have established a link between the dietary fiber intake (DFI) and post-stroke depression (PSD). Drawing on data collected in the National Health and Nutrition Examination Survey (NHANES) between 2005 and 2018, this investigation systematically examined the association between DFI and PSD in US women.

**Methods:**

A cross-sectional study was conducted using data from female participants in the NHANES from 2005 to 2018. The inclusion criteria comprised complete data on DFI, stroke history, and depression status. Multivariate logistic regression models were utilized to evaluate the association between DFI and the risk of PSD among the female population. To assess model validity, the Hosmer-Lemeshow test was performed to examine calibration, and a receiver operating characteristic (ROC) curve was constructed to measure discriminative ability. A restricted cubic spline (RCS) was employed to examine the correlations. Furthermore, subgroup analyses and interactions were also conducted to evaluate the stability of the relationship between DFI and PSD among different subgroups.

**Results:**

Among 13,143 screened female participants, 105 were diagnosed with PSD. The multivariate logistic regression model, after adjusting for all potential covariates, demonstrated that the odds ratio (OR) for the association between DFI and PSD was 0.92 [95% confidence interval (CI): 0.88–0.96; *p* < 0.001]. Model calibration was confirmed by the Hosmer-Lemeshow test (*p* = 0.549), and the area under the receiver operating characteristic curve (AUC) was 0.813 (95% CI: 0.775–0.852), indicating good model fit and strong discriminative ability. In the adjusted Model 3, when DFI was divided into quartiles, participants in the fourth quartile (Q4) exhibited a 70% lower risk of PSD compared to those in the first quartile (Q1) (OR: 0.30, 95% CI: 0.14–0.61; *p* = 0.001). The RCS analysis indicated an inverse association between DFI and the risk of PSD (*p* for non-linearity = 0.026). Subgroup analysis revealed that, except for subgroups stratified by age and body mass index (*p* < 0.05), there were no significant interactions between DFI and other specific subgroups (all interactions *p* > 0.05).

**Conclusion:**

The findings suggest a non-linear negative association between DFI and PSD risk among US women.

## 1 Introduction

Stroke, comprising ischemic and hemorrhagic types, is a prevalent acute cerebrovascular disorder. Globally, it ranks as the second leading cause of mortality, only surpassed by ischemic heart disease, and the third major contributor to disability (1). Approximately 66% of stroke patients suffer from limb dysfunction (2), significantly deteriorating both their quality of life and mental health. Consequently, they tend to develop secondary emotional disorders, such as depression and anxiety (3). Among the conditions following a stroke, depression is one of the most frequently observed. Approximately one-third of stroke survivors are diagnosed with post-stroke depression (PSD) (4). Moreover, the mortality risk for stroke patients with depression is 3.4 times higher than that of those without depression (5).

The manifestations of PSD are mainly low mood, sleep disorders, inattentiveness, significant changes in appetite or weight, low self-esteem, easy fatigue, and suicidal tendencies, usually lasting for more than 2 weeks (6). These symptoms can further impede the recovery of the patient’s neurological function, exacerbate disability, reduce their quality of life, worsen prognosis, and increase their mortality and recurrence rates. This imposes a heavy burden and harm on families and society. A meta-analysis has indicated that the incidence of PSD is notably higher in women than in men (7). Moreover, for stroke patients, female patients are more inclined to experience depressive symptoms than male patients, and the symptoms are more severe (8). Therefore, it is of utmost clinical significance to thoroughly investigate the pathogenesis associated with PSD and seek dietary factors associated with lower PSD risk, particularly among women.

Epidemiology has revealed a connection between dietary patterns, specific dietary factors, and depression (9). For instance, an increased intake of vegetables, fruit, soy products, and fish has been associated with a reduced risk of depression. Among these dietary components, dietary fiber, often referred to as the “seventh nutrient,” is a key factor. It is predominantly present in vegetables, fruit, nuts, grains, and whole-wheat foods and can be classified into soluble and insoluble types. The European Food Safety Authority and the US Food and Drug Administration define dietary fiber as all carbohydrates that are neither digested nor absorbed in the small intestine and possess a degree of polymerization of three or more monomeric units (10). Despite its indigestibility by the human body, incorporating dietary fiber into one’s diet offers numerous health benefits. It can facilitate regular defecation, help regulate blood sugar, lower blood lipid and cholesterol levels, contribute to weight management, and mitigate the risk of cardiovascular and cerebrovascular diseases (11). In addition, dietary fiber significantly affects the regulation of the richness, diversity, and stability of the gut microbiota (12). In fact, some studies have shown that there are significant differences in the gut microbiota between healthy individuals and female patients with depression (13, 14). The gut microbiota has the function of regulating the brain-gut axis, which is a communication channel between the brain and the gut (15). The occurrence and progression of PSD in women might be related to the dysregulation of the microbiota-gut-brain axis. Therefore, an increased dietary fiber intake (DFI) by women might be linked to a reduced risk of PSD.

Prior studies have delved into the connection between DFI and depression (16, 17). A clinical cross-sectional survey of the hypertensive population in China showed that DFI correlated negatively with the occurrence of post-hypertensive depression (16). Likewise, the National Health and Nutrition Examination Survey (NHANES) reported an association between DFI and depressive symptoms, indicating that a higher DFI was linked to a lower prevalence of such symptoms (17). A cross-sectional study of 12 to 18-year-old females in Iran indicated that the DFI of healthy females was significantly higher than that of depressive patients (18). Moreover, a study involving 1977 participants in Japan found that a higher intake of vegetables and fruit in the daily diet, meaning an adequate intake of dietary fiber, had a positive effect on preventing depression (9).

Despite an increasing amount of evidence indicating that DFI is closely associated with various health conditions, current research on the relationship between DFI and PSD, specifically in women, remains scarce. Consequently, we undertook a comprehensive cross-sectional survey using data from NHANES covering 2005 to 2018 to explore the association between DFI and PSD in women.

## 2 Materials and methods

### 2.1 Data sources

The NHANES is a comprehensive study carried out by the National Center for Health Statistics (NCHS), which operates under the Centers for Disease Control and Prevention in the United States. This database is used to assess the health and nutritional status of the US population. The NCHS Ethics Review Board has approved this study, and all participants have provided written informed consent (19). The survey has amassed a large volume of data through household interviews, physical examinations at the Mobile Examination Center, and laboratory tests overseen by highly trained medical professionals. More information about NHANES can be accessed at https://www.cdc.gov/nchs/nhanes/?CDC_AAref_Val.

**Figure 1:**
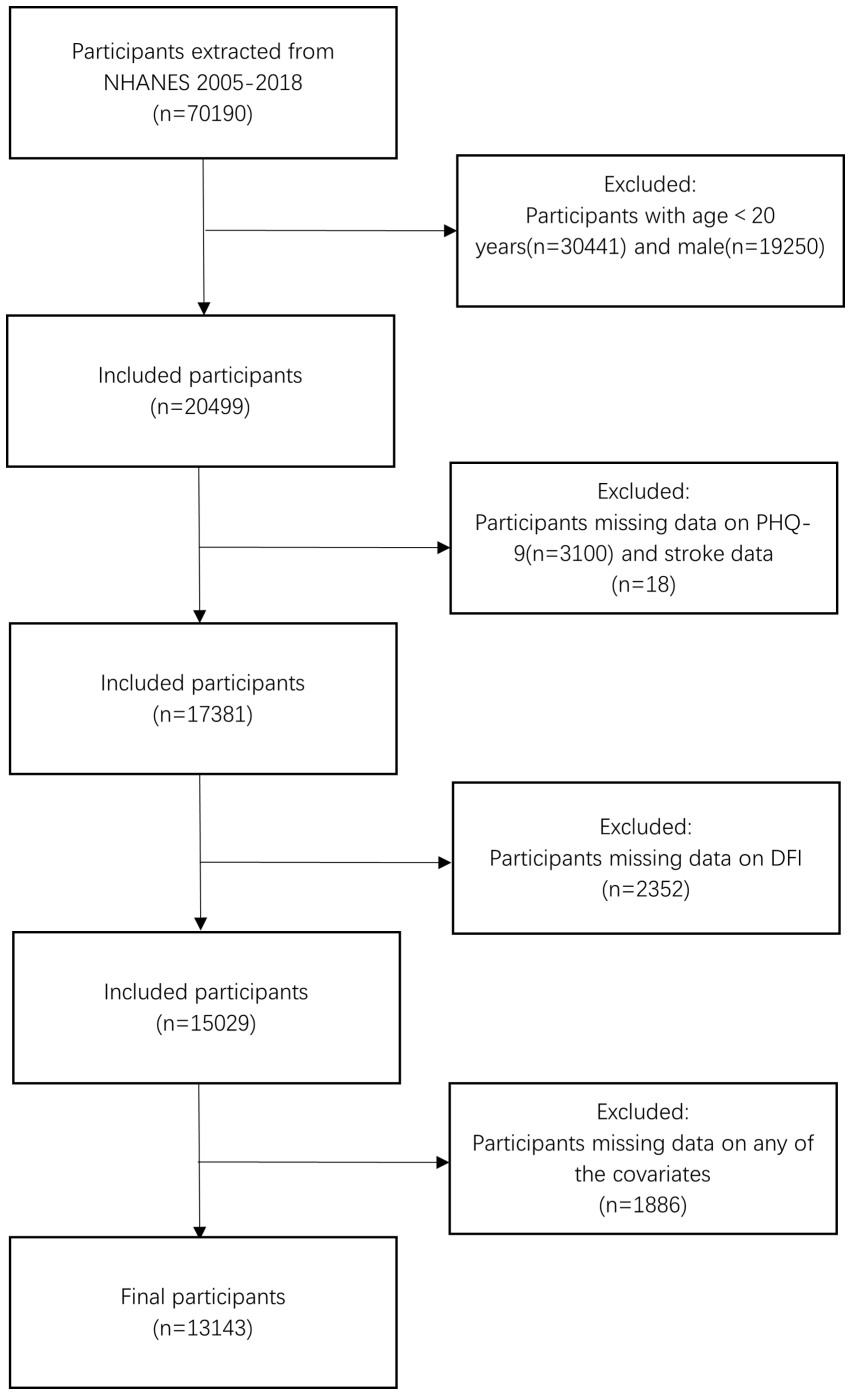
Flowchart of the sample selection from NHANES 2005–2018.

The present study was a cross-sectional observational study that included women aged 20 and older (*n* = 20,499) from seven cycles of NHANES spanning from 2005 to 2018. We excluded participants with missing data from the Patient Health Questionnaire-9 (PHQ-9) and stroke-related questionnaires (*n* = 3,118), those lacking DFI data (*n* = 2,352), and individuals without covariate information (*n* = 1886). Finally, 13,143 participants were included in the analysis. The detailed participant selection process is shown in Figure 1.

### 2.2 DFI

In NHANES, data on DFI were obtained by assessing dietary intake through 24-h dietary recall. All participants underwent two 24-h dietary recall interviews. The first questionnaire was administered at the Mobile Examination Center during the initial examination, and the second questionnaire was collected via telephone 3 to 10 days later. For dietary fiber data with no missing values in both interviews, the mean was calculated and used as the DFI for each participant. Detailed information about the dietary fiber data is available online at https://wwwn.cdc.gov/Nchs/Data/Nhanes/Public/2005/DataFiles/DR1TOT_D.htm#DR1TFIBE.

### 2.3 PSD

Stroke diagnosis was defined as a “yes” response to the question, “Has a doctor or other health professional ever told you that you had a stroke?” (20). The PHQ-9 scale was used to evaluate depression symptoms (21). The PHQ-9 ranges from 0 to 27, with higher scores indicating more severe depressive symptoms. According to previous research, depression is defined as a PHQ-9 score of ≥ 10 (22). In this study, participants were diagnosed with PSD if they had a history of stroke and scored 10 or higher on PHQ-9 (23). Notably, in NHANES, the PHQ-9 assesses depressive symptoms over the past 2 weeks. Our PSD diagnosis integrates a historical stroke diagnosis (via the self-reported question “Has a doctor or other health professional ever told you that you had a stroke?” at any time before the survey) and recent depressive symptoms (assessed by PHQ-9). While NHANES does not specify the time interval between stroke diagnosis and depression assessment—an interval varying among participants—this method reflects the clinical reality that PSD can emerge at diverse time points after a stroke, capturing the co-occurrence of a stroke history and recent depressive symptoms.

### 2.4 Covariates

To assess the potential impact of confounding factors, we selected several key potential covariates based on previous studies, including age, race, marital status, education level, body mass index (BMI), poverty-income ratio (PIR), energy intake, hypertension, diabetes, and hyperlipidemia. These covariates might be confounders associated with DFI and PSD in women. The age of the participants selected for this study refers to their age at the time of screening. Race was divided into four categories: Mexican Americans, non-Hispanic White, non-Hispanic Black, and individuals of other races. Marital status was divided into three groups: divorced/separated/widowed, married/living with a partner, and never married. Education level was classified into three groups: less than high school, high school or General Educational Development, and above high school. We determined BMI by dividing the participant’s weight (in kg) by the square of their height (in m). We classified BMI into three groups: normal (<25.0), overweight (25.0–30.0), and obese (>30.0) (24). These classifications adhere to clinical guidelines recommended by the World Health Organization (WHO), which are widely used in epidemiological research to standardize health risk assessment. This aligns with the established association between higher BMI and increased risks of cardiovascular diseases and metabolic disorders, which are relevant potential confounders for PSD. Household income was classified based on the PIR into three categories: PIR < 1.3, 1.3 ≤ PIR ≤ 3.5, or PIR > 3.5 (25). Energy intake (kcal/day) was derived from 24-h dietary recall data. Its calculation method is the same as that of the above-mentioned DFI (g/day). Hypertension was determined based on self-reported doctor-diagnosed results or blood pressure measured during physical examinations. Participants who met at least one of the following criteria were considered to have hypertension: (1) An average systolic blood pressure ≥ 140 mmHg; (2) average diastolic blood pressure ≥ 90 mmHg; (3) self-reported diagnosis of hypertension. Similarly, participants who met at least one of the following criteria were considered to have diabetes: (1) fasting plasma glucose ≥ 7.0 mmol/L; (2) glycated hemoglobin (HbA1c) ≥ 6.5 mmol/L; (3) self-reported doctor-diagnosed diabetes. Hyperlipidemia was diagnosed when any one of the following four criteria was satisfied: (1) having a Triglyceride level ≥ 150 mg/dL; (2) presenting a total cholesterol level ≥ 200 mg/dL; (3) showing a low-density lipoprotein level ≥ 130 mg/dL; (4) exhibiting a high-density lipoprotein level < 50 mg/dL.

### 2.5 Statistical analysis

Statistical analysis was performed using IBM SPSS Statistics V26 software and the Free Statistics analysis platform. A *p*-value < 0.05 was considered statistically significant. Continuous data were expressed as the mean ± standard deviation or interquartile range and compared using a *t*-test or non-parametric tests as appropriate. Categorical variables were presented as percentages (%) and compared using the chi-square test. For dietary fiber intake (DFI), quartile cut-off points (DFI ≤ 10.65, 10.66 ≤ DFI ≤ 14.45, 14.46 ≤ DFI ≤ 19.70, DFI ≥ 19.71) were determined in a data-driven manner. We sorted DFI values of all female participants and divided them into four equal parts based on the 25th, 50th, and 75th percentiles. This approach ensures a balanced distribution of intake levels across subgroups, facilitating detailed trend analysis of PSD risk as DFI increases. Multivariate logistic regression models were employed to evaluate the relationship between DFI (including continuous variables and quartile groups) and PSD. To assess model validity, we performed the Hosmer-Lemeshow test to examine calibration (non-significant *p* > 0.05 indicating good agreement between predicted and observed outcomes) and constructed a ROC curve to measure discriminative ability, calculating the AUC and its 95% confidence interval (CI). The ROC curve was generated by plotting sensitivity against 1-specificity across all classification thresholds. Three models were constructed. Model 1 was for crude analysis. And Model 2 was adjusted for demographic characteristics (age, race, marital status, education). Furthermore, Model 3 included additional covariates such as BMI, PIR, energy intake, hypertension, diabetes and hyperlipidemia (26).

Restricted cubic spline (RCS) analysis was employed to determine whether there was a negative non-linear relationship between DFI and the risk of PSD in women. Additionally, to gain a deeper understanding of the relationship between DFI and PSD among women in different subgroups, we conducted subgroup analyses and interaction analyses based on age, marital status, education level, PIR, BMI, and hyperlipidemia.

## 3 Results

### 3.1 Baseline characteristics of NHANES female participants (2005–2018)

Table 1 shows the baseline characteristics of NHANES female participants. A total of 13,143 female participants were included in this study, among whom 105 participants had PSD. The average age of the PSD group was (57.7 ± 12.2) years, which was higher than that of the non-PSD group, (49.2 ± 17.6) years. The DFI was divided into quartiles, namely four groups (DFI ≤ 10.65, 10.66 ≤ DFI ≤ 14.45, 14.46 ≤ DFI ≤ 19.70, and DFI ≥ 19.71). The risk of PSD in Q2–Q4 was significantly lower than that in Q1. Compared with participants without PSD, those with PSD were generally older, had a lower level of education, were divorced/separated/widowed, had a lower PIR, a higher BMI, a history of hypertension, diabetes, and hyperlipidemia, and a lower DFI intake (all *p* < 0.05).

**Table 1:** Characteristics of the study population from NHANES 2005–2018.

Variable	Overall (*n* = 13,143)	Non-PSD (*n* = 13,038)	PSD (*n* = 105)	*p*
Age (years), Mean ± SD	49.3 ± 17.6	49.2 ± 17.6	57.7 ± 12.2	<0.001
Race (%)				0.162
Mexican American	1981 (15.1)	1970 (15.1)	11 (10.5)	
Non-Hispanic White	5,970 (45.4)	5,920 (45.4)	50 (47.6)	
Non-Hispanic Black	2,726 (20.7)	2,697 (20.7)	29 (27.6)	
Other	2,466 (18.8)	2,451 (18.8)	15 (14.3)	
Education level (%)				0.013
Less than high school	2,740 (20.8)	2,710 (20.8)	30 (28.6)	
High school or GED	2,907 (22.1)	2,877 (22.1)	30 (28.6)	
Above high school	7,496 (57.0)	7,451 (57.1)	45 (42.9)	
Marital status (%)				0.009
Widowed/divorced/separated	3,625 (27.6)	3,582 (27.5)	43 (41)	
Married/living with partner	7,332 (55.8)	7,284 (55.9)	48 (45.7)	
Never married	2,186 (16.6)	2,172 (16.7)	14 (13.3)	
PIR (%)				<0.001
<1.3	4,145 (31.5)	4,086 (31.3)	59 (56.2)	
1.3–3.5	5,006 (38.1)	4,970 (38.1)	36 (34.3)	
>3.5	3,992 (30.4)	3,982 (30.5)	10 (9.5)	
BMI, kg/m^2^ (%)				0.018
<25	3,836 (29.2)	3,816 (29.3)	20 (19)	
25–30	3,727 (28.4)	3,700 (28.4)	27 (25.7)	
>30	5,580 (42.5)	5,522 (42.4)	58 (55.2)	
Energy (kcal), Mean ± SD	1755.7 ± 624.7	1756.2 ± 623.4	1685.2 ± 771.1	0.246
Hypertension (%)				<0.001
Yes	5,489 (41.8)	5,410 (41.5)	79 (75.2)	
No	7,654 (58.2)	7,628 (58.5)	26 (24.8)	
Diabetes (%)				<0.001
Yes	2043 (15.5)	2010 (15.4)	33 (31.4)	
No	11,100 (84.5)	11,028 (84.6)	72 (68.6)	
Hyperlipidemia (%)				0.04
Yes	8,781 (66.8)	8,701 (66.7)	80 (76.2)	
No	4,362 (33.2)	4,337 (33.3)	25 (23.8)	
Quartiles of DFI (gm) (%)				<0.001
Q1	3,945 (30.0)	3,893 (29.9)	52 (49.5)	
Q2	3,085 (23.5)	3,064 (23.5)	21 (20)	
Q3	3,045 (23.2)	3,026 (23.2)	19 (18.1)	
Q4	3,068 (23.3)	3,055 (23.4)	13 (12.4)	
DFI (gm), Median (IQR)	13.8 (9.8, 19.2)	13.9 (9.8, 19.2)	10.9 (7.0, 15.3)	<0.001

Mean ± SD for continuous variables, except DFI (gm), which is presented as Median (IQR); percentages (%) for categorical variables.

### 3.2 The correlation between DFI and PSD among women

The results of the multivariable logistic regression analysis for DFI and risk of PSD among women are presented in Table 2. The odds ratio (OR) of Model 1 was determined to be 0.93 (95% CI: 0.90–0.97, *p* < 0.001). This outcome suggested a negative association between the DFI, considered as a continuous value, and PSD. Upon accounting for all covariates within Model 3, the OR was calculated to be 0.92 (95% CI: 0.88–0.96, *p* < 0.001). Model fit was validated by the Hosmer–Lemeshow test (*χ*^2^ = 6.887, df = 8, *p* = 0.549), indicating good consistency between the predicted and observed values. This indicated that each 1 g/day increment in DFI was associated with 8% lower odds of PSD. Subsequently, the DFI was categorized into quartiles to investigate the relationship between the DFI and PSD. After making adjustments for potential confounding factors, when compared to individuals with low DFI values in the Q1 group, the adjusted ORs for the association between the DFI and PSD in the Q2, Q3, and Q4 groups were 0.50 (95% CI, 0.29–0.84, *p* = 0.009), 0.45 (95% CI, 0.25–0.80, *p* = 0.006), and 0.30 (95% CI, 0.14–0.61, *p* < 0.001), respectively. Compared with Q1, participants in Q2, Q3, and Q4 exhibited 50, 55, and 70% lower odds of PSD, respectively (adjusted OR: 0.50, 0.45, 0.30; all *p* < 0.001). Moreover, the *p*-value for trend was significant (*p* < 0.001).

**Table 2:** Logistic regression analysis of the correlation between DFI and the risk of developing PSD.

Exposure	Model 1	Model 2	Model 3
	OR (95% CI) *P*	OR (95% CI) *P*	OR (95% CI) *P*
DFI	0.93 (0.90–0.97)	0.94 (0.91–0.97)	0.92 (0.88–0.96)
*p*-value	<0.001	<0.001	<0.001
Categories			
Q1(DFI ≤ 10.65)	1 (Ref)	1 (Ref)	1 (Ref)
Q2(DFI 10.66–14.45)	0.51 (0.31–0.85)	0.52 (0.31–0.86)	0.5 (0.29–0.84)
	0.01	0.011	0.009
Q3(DFI 14.46–19.70)	0.47 (0.28–0.80)	0.49 (0.29–0.83)	0.45 (0.25–0.80)
	0.005	0.009	0.006
Q4(DFI ≥ 19.71)	0.32 (0.17–0.59)	0.36 (0.19–0.67)	0.3 (0.14–0.61)
	<0.001	0.001	0.001
*p* for trend	<0.001	<0.001	<0.001

OR, odds ratio; CI, confidence interval. Q1 serves as the reference group. Model 1 unadjusted. Model 2 adjusted for age, race, marital status and education level. Model 3 adjusted for all covariates including age, race, marital status, education level, BMI, PIR, energy intake, hypertension, diabetes and hyperlipidemia. The overall sample size is *n* = 13,143, and the exact sample sizes for each DFI quartile are shown in Table 1.

### 3.3 Non-linear associations and ROC curve

**Figure 2:**
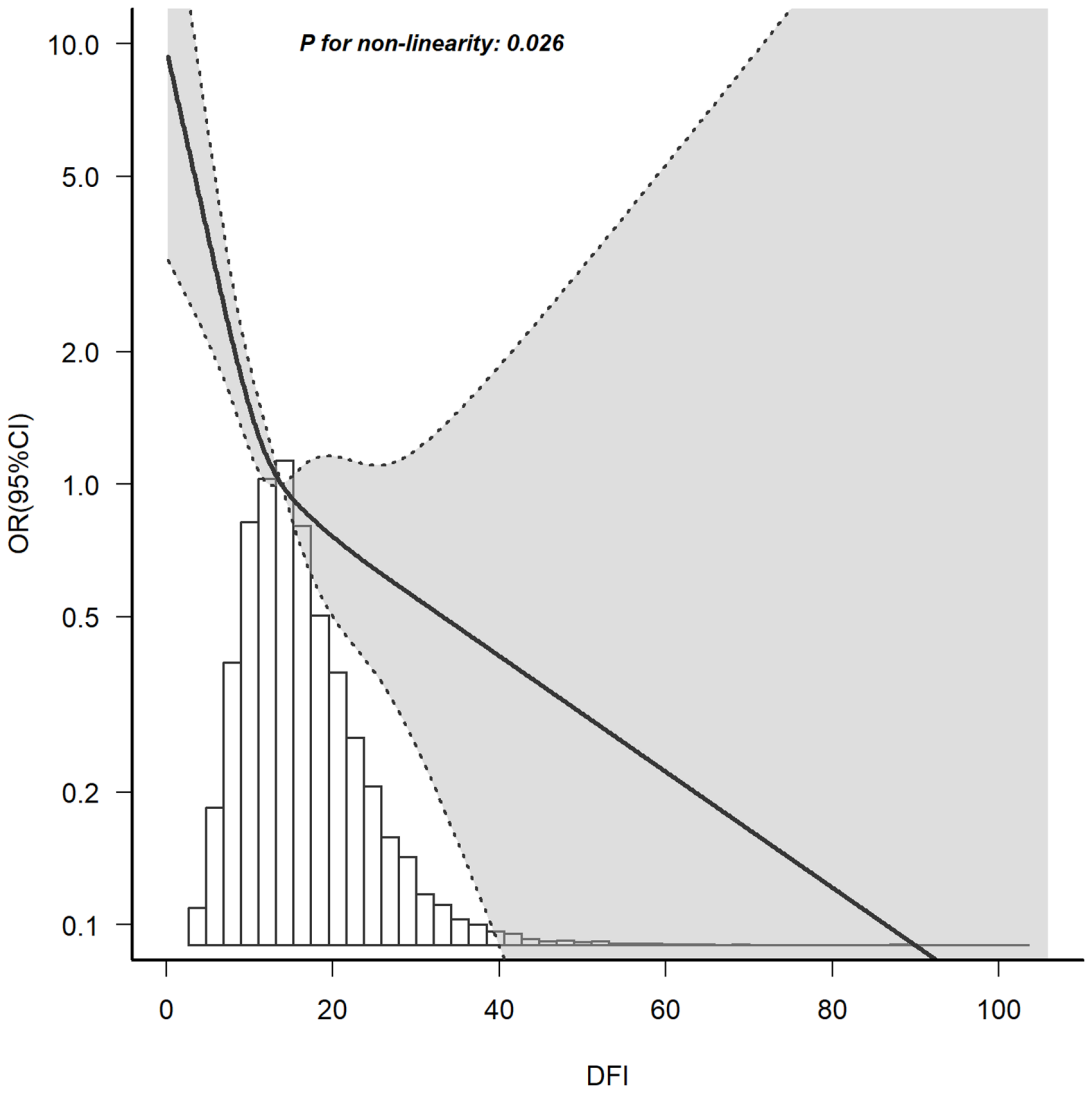
RCS plot of the association between DFI and the risk of PSD. A non-linear negative correlation was found between DFI and PSD. Solid and dashed lines represent the OR and its corresponding 95% confidence interval, respectively.

The relationship between DFI and the risk of PSD determined using RCS is shown in Figure 2. After adjusting for factors such as age, race, marital status, education level, BMI, PIR, energy intake, hypertension, diabetes, and hyperlipidemia, it was noted that a non-linear correlation exists, with DFI and the risk of PSD as the relevant variables (*p* for non-linearity = 0.026). The RCS analysis showed a trend of decreasing first and then leveling off. When DFI was less than around 20, as DFI increased, the OR of PSD decreased significantly. However, when DFI was greater than around 20, the rate of decrease in the OR lessened and tended to stabilize.

**Figure 3:**
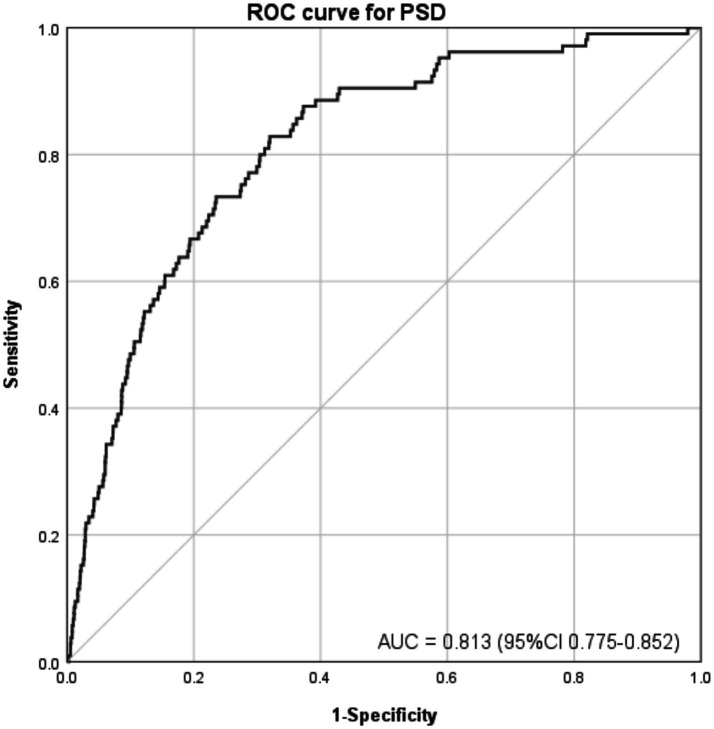
ROC curve for evaluating the predictive power of DFI in PSD. After adjusting all covariates, sensitivity was plotted against 1-specificity. The area under the curve (AUC) was 0.813 (95% CI: 0.775–0.852, *p* < 0.001), reflecting strong discriminative ability for predicting PSD.

The ROC curves were employed to assess the predictive efficacy of DFI (a continuous variable) for PSD after adjusting all covariates, as depicted in Figure 3. The area under the ROC curve (AUC) is proportional to the predictive value of the index; a larger AUC indicates higher predictive accuracy, while a smaller one suggests lower predictive capability. By plotting the ROC curve, the DFI index demonstrated an AUC of 0.813 (95% CI: 0.775–0.852, *p* < 0.001), highlighting the significant predictive utility of DFI in relation to PSD. This finding indicates that when all covariates are adjusted for, DFI serves as a meaningful predictor for PSD, enhancing the comprehensiveness of PSD risk assessment.

### 3.4 Subgroup analysis and interactions among women

**Figure 4:**
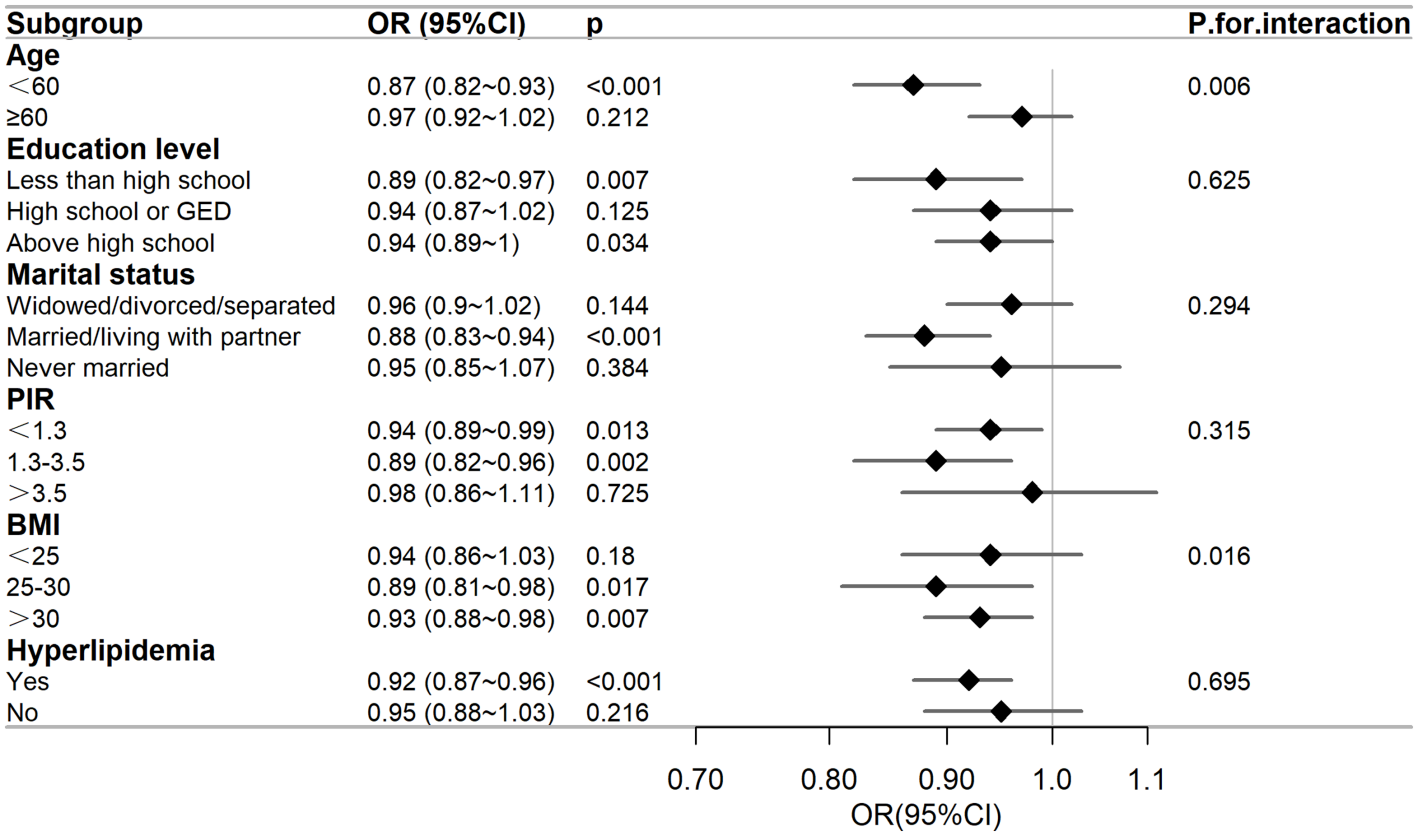
Subgroup analysis between DFI and the risk of PSD. The adjusted covariates were age, race, marital status, education level, BMI, PIR, energy intake, hypertension, diabetes, and hyperlipidemia. The figure shows that education level, marital status, PIR, and hyperlipidemia do not affect the negative correlation between DFI and the risk of PSD.

To determine whether the relationship between DFI and the risk of PSD was consistent across subgroups, we conducted subgroup and interaction analyses, and presented the results in a forest plot, as shown in Figure 4. The subgroups were classified by age, education level, marital status, PIR, BMI, and hyperlipidemia. The results of the subgroup analysis indicated that there was a negative correlation between DFI and the risk of PSD in subgroups of female individuals who were aged under 60 years, had an educational level below or above high-school, were married or living with a partner, had a PIR ≤ 3.5, had a BMI ≥ 25 kg/m^2^, and had a history of hyperlipidemia (*p* < 0.05). The interaction analysis showed that there were interactions between DFI and age, as well as between DFI and BMI (interaction *p* = 0.006, *p* = 0.016), while there was no interaction with the other variables (p for interaction > 0.05).

## 4 Discussion

In this study, we uncovered a negative relationship between DFI and PSD among women. This association held up and remained steadfast after controlling for possible confounding factors. Significantly, female participants with the highest DFI quartile had a 70% decreased probability of experiencing PSD as opposed to those with the lowest DFI quartile.

As far as we know, this was the first use of the NHANES database to analyze the connection between DFI and PSD in women. This study aimed to bridge the research gap by comprehensively evaluating the impacts of DFI on the incidence of PSD. After accounting for all confounding variables, RCS analysis indicated the presence of a non-linear relationship between DFI and the risk of PSD (p for non-linearity = 0.026). As illustrated in Figure 2, the OR of PSD decreased as the DFI increased. Additionally, a saturation effect was noted, with the indication being that the probability of PSD development is relatively low when DFI is at elevated levels. During the subgroup assessment, the inverse relationship between DFI and PSD in women appeared to be stronger among individuals under 60 years old. These individuals had an educational level either below or above high school, were married or living with a partner, had a PIR of 3.5 or less, a BMI equal to or greater than 25 kg/m^2^, and a history of hyperlipidemia. Interaction analysis revealed that this negative correlation was not modified by educational level, marital status, PIR, or hyperlipidemia (p for interaction > 0.05). This indicates that the association between DFI and PSD may remain unaltered by these elements. Subgroup analysis showed significant interactions for DFI with age (*p* = 0.006) and BMI (*p* = 0.016). For age, in those aged < 60 years, DFI’s protective effect was stronger (OR = 0.87, 0.82–0.93, *p* < 0.001). Biologically, younger individuals have better gut function (e.g., more short-chain fatty acid-producing bacteria), maximizing DFI’s mental health benefits (27). Chronic inflammation in older adults may weaken DFI’s protective effects (28, 29). Sociodemographically, younger individuals more readily maintain optimal DFI, whereas older adults often exhibit suboptimal DFI due to physiological declines such as masticatory dysfunction (30). Concurrently, they face stressors including loneliness and social isolation, which may interact with DFI (31). For BMI, in ≥25 kg/m^2^ groups (25–30: OR = 0.89, *p* = 0.017; > 30: OR = 0.93, *p* = 0.007), DFI’s effect was more evident. Biologically, higher BMI often brings metabolic issues (e.g., insulin resistance) and inflammation, disrupting gut-brain axis regulation (32). Sociodemographically, higher BMI may link to lower socioeconomic status, causing lower DFI intake and more stressors, affecting PSD risk (33). Nevertheless, the outcomes of the subgroup analysis merely represent an initial probe. Thus, a greater number of relevant studies are required to conduct further validation.

Previous studies have demonstrated that the gut microbiota has a substantial impact on decreasing the risk of depression in women (34–36). The gut and brain communicate through neural, endocrine, and immune pathways (37), with the gut microbiota acting as a key regulator in this process. Dietary fiber intake is linked to gut microbiota balance, which has been associated with lower depression risk through potential inflammatory and neurotransmitter-regulating pathways. The potential mechanisms through which dietary fiber alleviates depression in women can be summarized as follows. First, dietary fiber promotes the production of short-chain fatty acids. By providing energy for the gut microbiota, dietary fiber encourages the growth of beneficial bacteria (38, 39). These beneficial bacteria produce short-chain fatty acids as their main metabolites. Short-chain fatty acids have anti-inflammatory and antioxidant effects, which can positively affect the gut, protect the integrity of the intestinal barrier (10), and enhance intestinal permeability, collectively contributing to a reduced risk of depression or alleviation of depressive symptoms. Second, dietary fiber helps suppress inflammation. Research has shown a negative correlation between DFI and inflammation, and reducing inflammation is known to alleviate depression in women (40). A strong link between inflammation and depression has been well-established (41–43), with inflammation being a key risk factor for major depressive disorder (44). Dysregulation of the gut microbiota can lead to chronic inflammation and compromise the intestinal barrier (45). However, increased DFI has been shown to lower inflammatory markers, optimize gut microbiota composition, and improve the intestinal microenvironment (46). Additionally, dietary fiber can modulate intestinal pH and permeability, further reducing inflammation and thereby improving depressive symptoms (47). Third, dietary fiber might influence depression by regulating neurotransmitters. Although this mechanism is not yet fully understood, neurotransmitters are critical chemical messengers in the brain, playing a vital role in mood regulation (48). Dysregulation of neurotransmitters, such as serotonin and dopamine, is closely associated with depression. For example, serotonin is essential to regulate mood and sleep (49), and lower levels of its precursor, tryptophan, have been linked to higher depression scores (50). An animal study has shown that a high-fiber diet can enhance intestinal motility and increase serotonin expression in mice, alleviating depressive symptoms (51). Similarly, dopamine, a key regulator of mood, endocrine function, and memory (52), is often dysregulated in depression (53). Studies have consistently found lower dopamine levels in depressed individuals compared with those in healthy controls (54, 55). An animal study further demonstrated that dietary fiber supplementation could reduce neuroinflammation and elevate dopamine levels in depressed mice, improving depressive-like behaviors (56).

Beyond these mechanisms, dietary fiber might also impact female depression through other pathways. For instance, a meta-analysis suggested that fluctuations in estrogen levels significantly increase the risk of depression in women (57). In a study of 3,054 American women higher DFI was associated with fewer depressive symptoms in premenopausal women, suggesting that dietary fiber might help to regulate estrogen levels and thereby improve mood (58).

Although NHANES data do not differentiate between dietary fiber subtypes (e.g., soluble vs. insoluble), our findings of a non-linear negative association (Figure 2) and a 70% reduced risk of PSD in the highest DFI quartile (≥19.71 g/day, OR = 0.30, Table 2) suggest that increased total fiber intake is associated with mental health benefits. Future studies incorporating detailed dietary data to stratify fiber subtypes could further clarify their differential roles in PSD prevention.

These mechanisms collectively explain the observed negative correlation between DFI and PSD in women. It is important to note that the beneficial effects of dietary fiber on PSD are likely caused by the interplay of multiple factors rather than a single mechanism. However, further research is needed to fully elucidate these complex interactions.

While conclusive evidence supporting the use of DFI in treating PSD in women is still lacking, the observed association suggests that higher DFI could be a potential marker for PSD risk stratification in clinical settings, pending confirmation by longitudinal studies. For women with PSD, a higher DFI might have a positive impact on their condition. Therefore, regular monitoring of DFI could aid in the early detection of depressive symptoms, providing a foundation for early intervention and improved patient outcomes. In addition to the benefits of DFI, it is essential to actively screen stroke survivors for physical and mental health issues and address them promptly. Integrating DFI into the assessment and management of PSD in women could further enhance treatment efficacy.

Our findings highlight a negative correlation between DFI and PSD in women, suggesting that higher DFI could be a modifiable factor associated with lower PSD prevalence. This research underscores the importance of promoting higher DFI as a potential strategy to lower the risk of PSD in women. It is crucial to recognize that excluding individuals with missing data during the data curation process might introduce selection bias. If the missing data were systematically related to both DFI and PSD–for instance, individuals with severe PSD might be less likely to complete the survey–the observed association between DFI and PSD could be compromised. For example, if participants with higher DFI and better mental state (owing to milder PSD symptoms) were over-represented in the dataset, our results might overestimate the protective role of DFI. On the contrary, if individuals with low DFI and severe PSD were under-represented because of missing data, the actual association might be underestimated. Moreover, although our multivariate model adjusted for several variables, residual confounding from unmeasured factors remains a concern. For instance, medication use, such as antidepressants or other psychotropic drugs, which are not recorded in NHANES, could influence both DFI and PSD risk. NHANES only records whether a stroke occurred but lacks detailed measures of stroke severity, which might affect both dietary patterns (potentially lowering DFI in more severe cases) and PSD risk. While NHANES does contain data on dietary supplement use, we did not systematically incorporate it into our analysis, and such use could impact DFI and depression risk. These unmeasured confounders highlight the need for future studies to more comprehensively assess these factors to better clarify the association between DFI and PSD. Firstly, due to the cross-sectional design of NHANES, it was infeasible for us to determine a cause-and-effect link between DFI and PSD. Future research should include longitudinal studies to clarify this relationship. Secondly, PSD was assessed through self-reports in NHANES, which might have introduced biases because of individual differences. The use of PHQ-9 for depression assessment also differs from clinical diagnostic methods. Additionally, self-reported dietary data might be subject to recall bias, potentially affecting the accuracy of DFI estimates. Another limitation is the lack of stroke subtype classification in NHANES. The dataset records self-reported stroke history without distinguishing between ischemic and hemorrhagic types, which may have distinct pathological mechanisms affecting PSD. This precludes subtype-specific analyses and potentially masks heterogeneous associations between dietary fiber and depression risk across different stroke etiologies. Future studies with validated clinical or imaging-based subtype data are needed to clarify these differences. Finally, the study was restricted to US women. As a result, whether the results can be generalized to other populations is yet to be validated.

## 5 Conclusion

We discovered a non-linear negative association between DFI and the prevalence of PSD in US women. These results provide new insights into the role of a high-fiber diet in PSD risk assessment. To further validate our results, more randomized controlled trials or cohort studies are required.

## Data Availability

The original contributions presented in the study are included in the article/supplementary material, further inquiries can be directed to the corresponding author.
